# Integrated Microcantilever for Joint Thermal Analysis of Trace Hazardous Materials

**DOI:** 10.3390/s25103004

**Published:** 2025-05-09

**Authors:** Yuhang Yang, Xinyu Li, Zechun Li, Ming Li, Ying Chen, Shaokui Tan, Haitao Yu, Pengcheng Xu, Xinxin Li

**Affiliations:** 1 State Key Laboratory of Transducer Technology, Shanghai Institute of Microsystem and Information Technology, Chinese Academy of Sciences, Shanghai 200050, China; yuhangyang98@mail.sim.ac.cn (Y.Y.); zechunli@mail.sim.ac.cn (Z.L.); liming01@mail.sim.ac.cn (M.L.); chenying@mail.sim.ac.cn (Y.C.); tanshaokui@mail.sim.ac.cn (S.T.); yht@highend-mems.com (H.Y.); 2 University of Chinese Academy of Sciences, Beijing 100049, China; 3 Shanghai Aerospace Electronic and Communication Equipment Research Institute, Shanghai 201109, China; serene_li@163.com

**Keywords:** MEMS, resonant cantilever, TGA, DTA, thermal analysis

## Abstract

During the thermal analysis of hazardous materials, the thermal instruments available may face the risk of contamination within heating chambers or damage to the instruments themselves. Herein, this work introduces an innovative detection technology that combines thermogravimetric and differential thermal analysis with an integrated MEMS cantilever. Integrating polysilicon thermocouples and a heat-driven resistor into a single resonant cantilever achieves remarkable precision with a mass resolution of 5.5 picograms and a temperature resolution of 0.0082 °C. Validated through the thermal analysis of nylon 6, the cantilever excels in detecting nanogram-level samples, making it ideal for analyzing hazardous materials like ammonium perchlorate and TNT. Notably, it has successfully observed the evaporation of TNT in an air atmosphere. The integrated MEMS cantilever detection chip offers a groundbreaking micro-quantification solution for hazardous material analysis, significantly enhancing safety and opening new avenues for application.

## 1. Introduction

Thermal analysis is a crucial technology that examines how a substance’s intrinsic properties relate to temperature or time while under heating conditions. It is essential in fields such as chemical production [1,2,3], food safety [4,5,6], and drug testing [7,8,9], as it offers critical material characterization. Key thermal analysis techniques, including thermogravimetric analysis (TGA) and differential thermal analysis (DTA), provide valuable insights into material measurement and characterization. TGA monitors real-time mass changes during heating, revealing characteristics of thermal decomposition and composition [10,11], while DTA measures temperature differences between samples and reference materials to identify thermal effects related to phase changes and decomposition [12,13]. By combining TGA and DTA, researchers can gain a comprehensive understanding of the interactions between weight and heat, enhancing data reliability and addressing the limitations of traditional analysis methods [14,15]. The integrated TGA-DTA approach clarifies physical and chemical processes of materials during the heating process and facilitates a systematic examination of crucial parameters, such as thermal stability and multi-component composition [16].

The rapid advancement in material research clearly reveals the limitations of traditional thermal analysis instruments, which often require milligram quantities of samples [17]. The large sample size requirement significantly restricts their application in trace measurements and poses serious safety risks when testing flammable substances. Additionally, using a large sample can result in thermal hysteresis and uneven heating, ultimately compromising test efficiency. In response to these challenges, thermal analysis technology is evolving, and innovative solutions are emerging. Cantilevers have a wide application in the weighing of small masses [18]. Our group’s cantilever–TGA system can measure nanogram-level samples through frequency changes [19,20], although it does not include integrated temperature measurement for precise calorimetry. On the other hand, Mettler–Toledo’s MEMS DTA system offers calorimetry capabilities for microgram-level samples but fails to monitor mass changes in materials simultaneously, which hinders quantitative assessments [21,22]. Currently, MEMS thermal analysis technology is limited to isolated TGA or DTA measurements and has not yet achieved integrated joint detection on a chip. This gap results in insufficient temporal and spatial correlation between mass and thermal change data, highlighting the need for more robust solutions in the thermal analysis field.

Herein, we integrated thermocouples into a resonant cantilever for thermogravimetric measurements. Our innovation led to the development of a cantilever chip for the joint characterization of polymers and explosives through TGA-DTA. With the integrated thermocouples, the resonant cantilever retained its capabilities for cantilever–TGA testing while also enabling DTA measurements of the samples. Using this cantilever chip, which features an integrated thermopile, we constructed a measurement setup for simultaneous TGA-DTA characterization. This setup allows us to test trace samples at the nanogram level. Following calibration, the cantilever chip demonstrated a mass resolution of 5.5 picograms and a temperature resolution of 0.0082 °C. We applied MEMS thermal analysis technology to perform TGA-DTA tests on the decomposition of polymers, validating the efficiency and performance of our testing system. Additionally, we used this measurement device for TGA-DTA analysis on substances with explosive properties, such as ammonium perchlorate and TNT, to underscore the substantial potential application value of our thermal analysis systems in assessing hazardous and energetic materials, thereby advancing thermal analysis technology toward a chip-based approach.

## 2. Integrated Cantilever Chip Structure, Fabrication, and Thermal Analysis Measurement Setup

**Figure 1:**
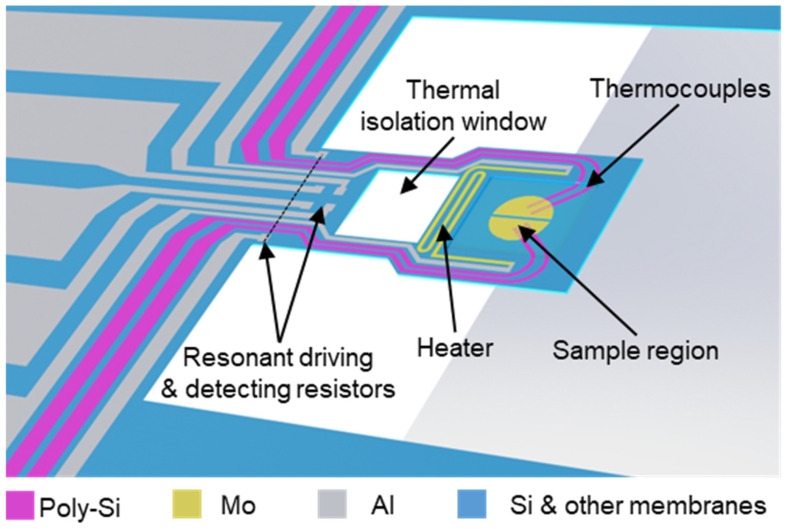
Structure of the thermocouple-integrated resonant cantilever.

As shown in Figure 1, the main component of the thermocouple-integrated resonant cantilever is a mass-type MEMS sensor, which measures sample mass changes based on the proportional relationship between the cantilever’s tiny mass and its resonant frequency [23]. At the base of the cantilever, a thermal drive resistor and a Wheatstone bridge are integrated for resonant driving and frequency detection. Two pairs of polysilicon thermocouples extend from the silicon substrate (the cold end) to the center of the sample area (the hot end). Utilizing the Seebeck effect, the output voltage of the thermocouples is proportional to the temperature difference between the cold end and the hot end [24,25], allowing for the detection of sample temperature information and the temperature difference signal.

**Figure 2:**
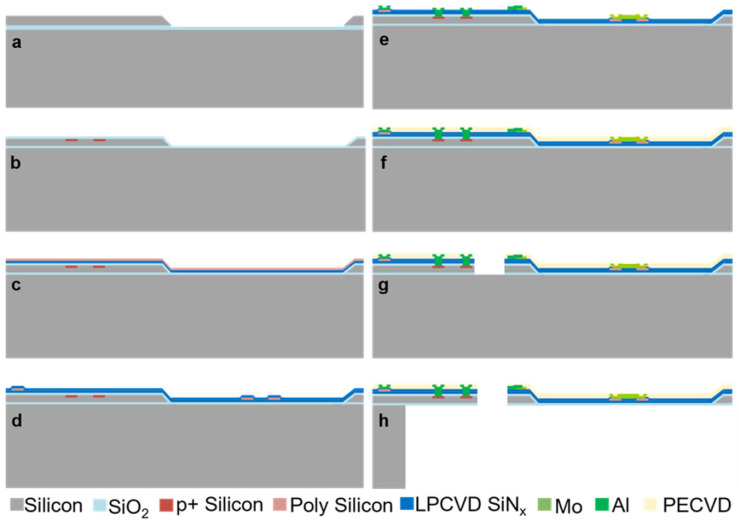
Fabrication process of the integrated cantilever chips. (**a**) Sample loading region fabrication. (**b**) Ion-implantation. (**c**) LPCVD SiNx and polysilicon deposition. (**d**) Fabrication of thermocouples. (**e**) Patterning of Mo heater and Al electric connection. (**f**) PECVD film deposition for antioxidant protection. (**g**) DRIE to pattern the shape of the cantilever. (**h**) DRIE from back to release the cantilever structure.

To control the thickness of our cantilever, our devices are fabricated on 4-inch (100) SOI wafers with a device layer thickness of 3 μm, a handle layer thickness of 500 μm, and a box layer thickness of 700 nm. The detailed fabrication process is shown in Figure 2: (a) The thermal SiO_2_ of 350 nm is fabricated through a heating oxidation furnace. After photolithography and a Reactive Ion Etching (RIE) etching of SiO_2_, a pool is defined by KOH wet etching for the sample loading region. (b) The silicon resistors of the resonant excitation and readout resistors (Wheatstone bridge) are defined by photolithography, ion implantation, and diffusion. (c,d) After depositing 200 nm SiNx and 500 nm polysilicon films by LPCVD, the main part of the polysilicon thermocouples is fabricated sequentially through ion implantation, diffusion, and RIE. A 300 nm SiNx film is then deposited to protect the thermocouples. (e) After etching the connection window of the poly-silicon thermocouple and silicon resistors, Mo metal films are patterned by the lift-off process to create microheaters and connections between thermocouples. Then, the Al metal films are patterned by the lift-off to form the electrical connections of silicon resistors. (f) Then, 200 nm PECVD SiO_2_/Si_3_N_4_ multi-films are deposited to provide antioxidant protection and electric isolation. (g) An RIE process of dielectric layers is followed by the DRIE of the device layer and the RIE of the box layer to form the shape of the resonant cantilever. (h) Then, the cantilever is released by removing the handling layer with the DRIE process from the back.

**Figure 3:**
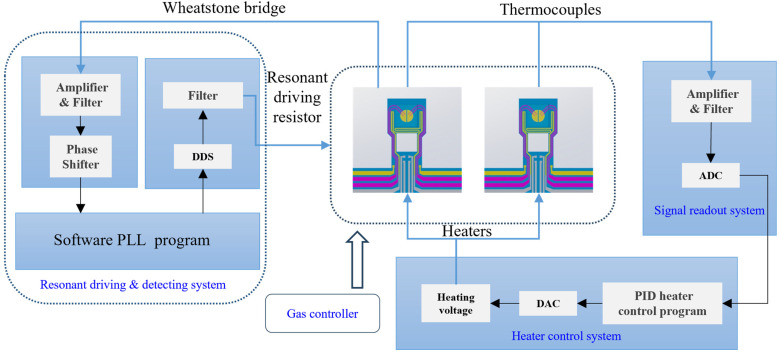
Illustration of the thermal analysis measurement setup featuring the integrated cantilever as the core TGA-DTA joint analysis chip.

Figure 3 illustrates the chip-based TGA-DTA thermal analysis system. The cantilever chip is positioned under an optical microscope to enable the real-time observation of changes in sample morphology during the heating process. Two chips are connected to the system: one is designated as the sample cantilever, which loads the sample, while the other, referred to as the reference cantilever, remains empty. This design minimizes common-mode noise interference. The resonant excitation and readout resistors of the sample beam are linked to the resonant detection PLL circuit to monitor the resonant frequency. The sampling frequency of the PLL circuit is 100 Hz. The driving voltage, U, applied to the driving resistor combines a DC signal with an AC signal, and the heating power is determined using the following equation:

(1) $$P\left(t\right)=\frac{{\left(U_{dc}+U_{ac}\right)}^{2}}{R}=\frac{2U_{dc}U_{ac}\cos\omega t+{U_{dc}}^{2}+0.5{U_{ac}}^{2}+0.5{U_{ac}}^{2}\cos2\omega t}{R}$$

where *U_dc_* represents the DC signal, *U_ac_* stands for the AC signal, and *ω* denotes the angular frequency [26]. By modifying the angular frequency of the AC signal, we can adjust the frequency of the driving signal. The output voltage from the thermocouples on the two cantilevers is connected to a signal readout circuit, which monitors and controls the temperature of the sample area. In order to realize the high-precision, low-noise detection of thermocouple signals, a corresponding low-noise signal amplification and reading circuit is designed based on instrumentation amplifiers. The heating drive circuit employs PID control to regulate the voltage of the heating resistor based on the user-defined temperature and the output from the thermocouple. This system enables precise programmed temperature control. Both circuits are linked to a computer to facilitate data recording and subsequent analysis.

To load the sample onto the device, we utilize a micromanipulator (Eppendorf, model PatchMan NP2). The sample is first dispersed in an organic solvent to form a solution or suspension. The microneedle equipped in the micromanipulator then draws up a specific volume of the liquid, allowing for the precise placement of the sample in the designated area under the microscope. Once positioned, the solution is evaporated, leaving the sample ready for testing.

## 3. Experimental Section

### 3.1. Materials and Chemical

The MEMS-integrated cantilever chips were batch-fabricated on silicon-on-insulator (SOI) wafers, featuring a 3 µm device layer, a 0.7 µm buried oxide layer, and a 400 µm silicon substrate. Nylon-6, ammonium perchlorate (AP), and trinitrotoluene (TNT) were purchased from Sigma-Aldrich Co., Ltd., Shanghai, China. Polystyrene (PS) microsphere samples were obtained from Huge Biotechnology Co., Ltd., Shanghai, China. During the test, nylon-6 and TNT were prepared as powders and dispersed in ethanol to form a suspension for sample loading, while AP was dissolved in water to form an aqueous solution. After loading, the cantilever was placed in a thermostatic drying oven, and the solvent was evaporated, leaving the sample ready for testing.

### 3.2. Instrumentation

Surface morphology characterization of the cantilever was performed using a scanning electron microscope (SEM, model SNE-4500M Plus, SEC, Republic of Korea) A non-contact infrared thermal imager (Fluke TiX560, Fluke, WA, America) with a spatial resolution of 20 μm was used to calibrate the working temperature. To compare the thermal analysis results between the MEMS thermal analysis chip and traditional instruments, a conventional thermogravimetric analyzer (Netzsch STA-449-F3, Netzsch, German) was used. This instrument enables thermal analysis of milligram-level samples within a temperature range from room temperature to 1000 °C, with a heating rate of up to 40 °C/min.

### 3.3. Measurement Process

Prior to use, the MEMS cantilever chip underwent a preconditioning process. The heating resistor voltage was adjusted to elevate the temperature of the sample region above 500 °C and maintained for 20–30 min. This step served to improve the Ohmic contact of the heater and to eliminate potential organic contaminants from the sample region. Before testing, the chip was placed in a thermostatic drying oven at 80 °C for 30 min to remove condensed moisture and other impurities that might affect thermal analysis. It is worth noting that, like crucibles, each chip is typically used to measure only one type of sample to avoid cross-contamination.

The thermal analysis procedure of our cantilever chip included baseline acquisition and signal measurement. For thermogravimetric analysis, a bare cantilever was first connected to the testing system. Environmental parameters such as ambient temperature, gas composition, and flow rate were set accordingly. A phase-locked loop (PLL) circuit was employed to track the resonant frequency. Once the frequency stabilized, the temperature of the sample region was controlled according to a predefined temperature program. The resonant frequency was synchronously recorded using the PLL circuit, and the resulting frequency–temperature–time data were stored as the baseline. For differential thermal analysis, thermocouple output was monitored using a dedicated signal acquisition circuit. After the thermocouple signal stabilized, the same temperature program was applied to the sample region. The output signals from the sample and reference beams, as well as their differential, were recorded to establish the DTA baseline. Subsequently, after loading the sample, the above steps were repeated under the same temperature control program. The measured signals were collected for both TGA and DTA modes. The heating rate is a crucial parameter in thermal analysis. Generally, a faster heating rate can enhance the intensity of the DTA signal; however, it may also result in greater measurement errors between TGA signals and temperature readings. Therefore, selecting an appropriate heating rate is essential. In this study, a heating rate of 5 °C/s was chosen based on this trade-off.

Further processing of the signals yields the desired thermal analysis curves. The baseline resonant frequencies of the cantilever beam at room temperature and at a high temperature, *T*, are denoted as *f*_00_ and *f*_0*T*_, respectively, while the resonant frequencies measured under actual signal conditions are *f*_10_ at room temperature and f_1T_ at temperature *T*. According to the resonant frequency–mass relationship, the ratio of the sample mass at temperature *T* (Δ*m_T_*) to the sample mass at room temperature (Δ*m*_0_) can be expressed as

(2) $$\frac{{\Delta m}_{T}}{{\Delta m}_{0}}=\frac{\frac{1}{f_{1T}^{2}}-\frac{1}{f_{0T}^{2}}}{\frac{1}{f_{10}^{2}}-\frac{1}{f_{00}^{2}}}$$

Thus, by processing the frequency information, the variation in sample mass—that is, the TGA curve—is obtained.

For the DTA signal, based on the Seebeck effect, the difference in the thermocouple outputs between the sample beam and the reference beam can be represented as

(3) $$∆V=\alpha \left({∆T}_{heat}+∆T_{loading}\right)$$

where *α* is the Seebeck coefficient of our chip, Δ*T_loading_* is the temperature differential induced by the sample, and Δ*T_heat_* represents the temperature difference generated by the heater due to inherent disparities in chip parameters (such as the heating resistor, heat capacity, and thermal conductivity). Therefore, by subtracting the baseline signal from the measured signal, the temperature difference caused by the sample is isolated, yielding the DTA curve.

## 4. Chip Characterization and Calibration

**Figure 4:**
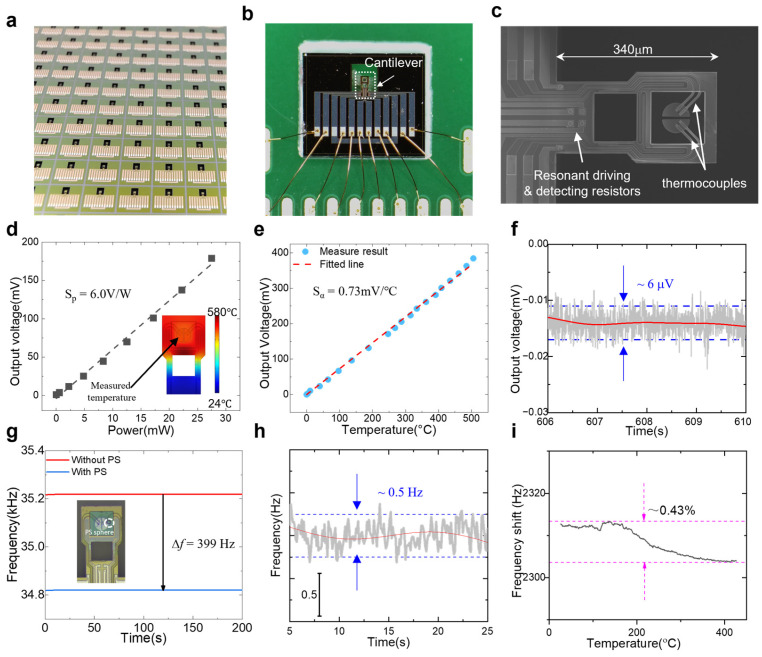
Characteristics and intrinsic performance of the integrated cantilevers. (**a**) Cantilever fabricated from a SOI wafer. (**b**) Cantilever packaged on a PCB for thermal analysis measurements. (**c**) SEM image of the cantilever. (**d**) Power sensitivity of the cantilever. (**e**) Temperature sensitivity of the cantilever. (**f**) Noise floor of the thermocouple output signal. (**g**) Mass sensitivity of the cantilever. (**h**) Noise floor of the frequency signal. (**i**) Frequency change in the cantilever during heating.

Figure 4a–c illustrate the morphology of the integrated cantilever chip, which serves as the core of the thermal analysis system. Figure 4a presents an optical image of the cantilever chip, manufactured in batches on a silicon-on-insulator (SOI) wafer. After laser scribing, each individual chip measured 2.3 mm in length and 2.7 mm in width. Figure 4b shows the chip affixed to a printed circuit board (PCB), resulting in a testing unit that can be easily connected to the test device. Figure 4c displays a scanning electron microscope (SEM) image of the cantilever beam structure, which has a length of 340 μm, a width of 190 μm, and a thickness of 3 μm. Additionally, the sample sink is settled on a 2 μm thick laminate film, with a diameter of 60 μm, while the thermal isolation window measures 100 μm by 90 μm.

We calibrated the thermal analysis performance of our test system using a non-contact infrared thermal imager with a spatial resolution of 20 μm to measure the temperature in the cantilever sample area. Simultaneously, the test system recorded parameters such as heating power and thermocouple output voltage at various temperatures. Figure 4d illustrates the relationship between thermocouple output voltage and heating power, demonstrating strong consistency. The inset in Figure 4d shows the finite element simulation results for the cantilever beam surface temperature distribution. The power response sensitivity parameter, obtained through linear fitting, indicates that the device’s power response reaches 6.0 V/W, which is three orders of magnitude higher than traditional thermal power detection instruments, which operate at the mV/W level [27,28]. Figure 4e presents the output voltage of the thermocouple at different hot junction temperatures, showing a linear response to temperature, in alignment with the Seebeck effect theory. After extracting parameters, we found that the Seebeck coefficient of the two pairs of polysilicon thermocouple structures reached 0.73 mV/K, highlighting the significant advantages of polysilicon thermocouple materials. Figure 4f shows the noise level of the thermocouple output signal, as read by the test system at room temperature. The noise produced by the two pairs of thermocouples at room temperature was measured at 6 μV, resulting in an equivalent noise temperature difference of 8.2 mK.

After that, we calibrated the mass detection performance of our chip. A flowing DC-biased AC was applied to the excitation resistor, and the Wheatstone bridge was also connected to the PLL interface circuit. In this way, we could measure the real-time frequency shift. By loading a standard polystyrene (PS) ball (diameter: 20 μm; density: 1.05 g/cm^3^) onto the cantilever beam, we were able to record the corresponding resonant frequency drift at room temperature after the frequency became stable. As depicted in Figure 4g, after loading a single PS ball, the measured change in resonant frequency was 399.0 ± 0.5 Hz, leading to a calculated mass response rate of 0.090 Hz/pg for the cantilever. Additionally, Figure 4h shows that the resonant frequency of the cantilever was tracked using a phase-locked loop (PLL) circuit, with a noise level of 0.5 Hz, which is in the same order of magnitude as the reported results [19]. Consequently, the mass resolution of our instrument reached 5.5 pg. The experimental results indicate that the mass resolution of our detection system is six orders of magnitude higher than that of traditional thermogravimetric analyzers, thereby holding the potential for the quantitative detection of nanogram-level samples. Silicon powder is an inert substance, and its mass remains constant with temperature changes. Figure 4i demonstrates the resonant frequency drift caused by the same silicon powder sample at increasing temperatures, which was induced by an integrated micro-heater. We observed that the frequency difference varied by only 0.43% when the temperature changed from room temperature to 400 °C. We conclude that the impact of temperature on the cantilever chip’s mass sensitivity is negligible. Table 1 shows the relevant parameters of our work compared to reported MEMS chips.

## 5. Joint TGA-DTA Characterization of the Decomposition of Nylon 6 Standard Samples

**Figure 5:**
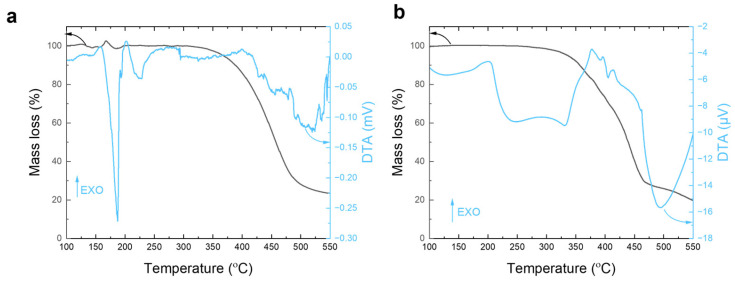
Comparative experimental results for nylon 6 standard samples obtained using two instruments: (**a**) the MEMS chip-based thermal analysis system and (**b**) the commercial Netzsch TGA-DTA instrument (model: STA-449-F3).

To validate the joint characterization capability of TGA-DTA, we conducted a comparative experiment to analyze the thermal decomposition characteristics of nylon 6 standard samples [31,32]. We utilized both our developed MEMS chip-based thermal analysis system and a traditional TGA-DTA instrument (model: Netzsch STA-449-F3, Netzsch, German. All experiments were performed in an air atmosphere. The MEMS system facilitated dynamic thermal analysis within a temperature range of 100–550 °C, achieving a fast heating rate of 5 °C/s. In contrast, the traditional instrument in our laboratory operates at a conventional test rate of only 0.33 °C/s (approximately 20 °C/min), which makes the MEMS system 15 times more efficient. The TGA-DTA curves from both experiments are compared in Figure 5.

The TGA curve obtained from the MEMS thermal analysis system, as shown in Figure 5, demonstrates a significant mass loss in a range of 350 °C to 500 °C. This loss corresponds to the high-temperature thermal decomposition process of the nylon 6 sample, resulting in a total mass loss of 74%. Notably, even though the MEMS test system operates in fast test mode at a heating rate of 5 °C/s, its TGA curve closely resembles that obtained by a commercial instrument operating at a much slower rate of 0.33 °C/s. In the DTA curve, the nylon 6 exhibits a sharp endothermic peak at 180 °C, indicating its melting process. The endothermic peak observed between 350 °C and 500 °C can be attributed to the high-temperature decomposition of nylon 6, which results in the release of gaseous products. Compared to traditional instruments, the MEMS test system demonstrates excellent consistency in test results, even with a heating rate 15 times higher. This confirms both the high efficiency and performance of our test system. This comparative experiment validates the reliability of the MEMS thermal analysis system under rapid scanning conditions. Its significant heating rate advantage enhances thermal analysis efficiency, offering a new technical approach for the rapid characterization of polymer materials. Additionally, it confirms that the measurements obtained from the MEMS thermal analysis system are reliable and applicable in various contexts.

## 6. Combined TGA-DTA Characterization of Hazardous Materials Using the Integrated Cantilever Chip

When using traditional thermal analysis instruments to measure samples that are explosive, corrosive, or strong oxidizers, the large sample volume and uneven temperature can increase the risk of instrument contamination or even damage. In contrast, our resonant cantilever beam requires samples at the nanogram (ng) level, which significantly reduces the risk of violent explosions or the accumulation of hazardous chemicals in the furnace chamber. As a result, our instruments offer considerable advantages for analyzing hazardous substances that pose potential health and safety risks.

**Figure 6:**
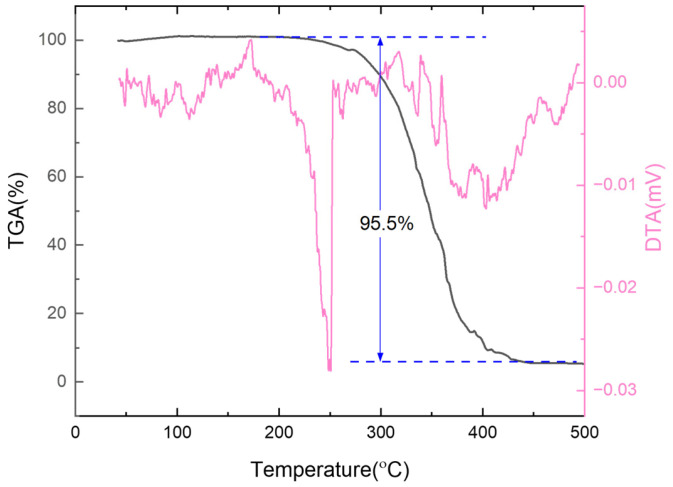
TGA-DTA characterization of the strong oxidant ammonium perchlorate (AP).

We began by measuring the thermal properties of a common strong oxidant, ammonium perchlorate (AP) [33,34]. Using a cantilever chip, we heated the sample from room temperature to 500 °C at a rate of 10 °C per second in an air atmosphere. The resulting TGA and DTA curves are illustrated in Figure 6. At 240 °C, ammonium perchlorate experiences a morphological transition from an orthorhombic phase to a cubic phase, which appears as an endothermic peak on the DTA curve. Between 280 °C and 400 °C, AP decomposes dramatically, resulting in a substantial mass loss of 95.5% on the TGA curve. The results indicate that ammonium perchlorate rapidly breaks down into gaseous products.

**Figure 7:**
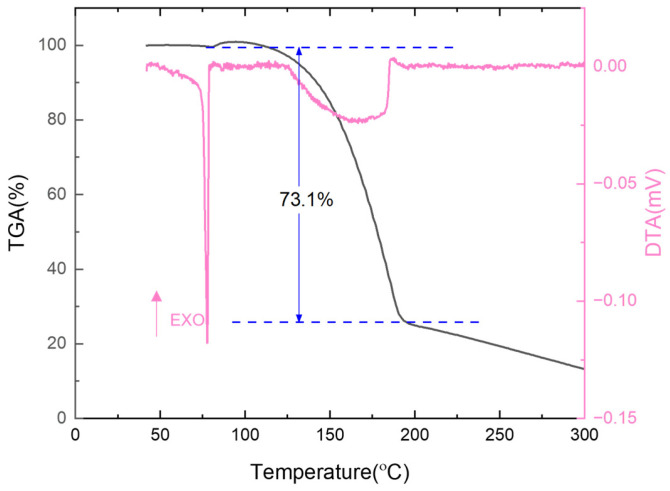
TGA-DTA combined characterization of explosive trinitrotoluene.

We further selected trinitrotoluene (TNT), a well-known explosive, for thermal analysis experiments [35,36]. We heated it from room temperature to 300 °C in an air atmosphere while simultaneously measuring the TGA and DTA curves. The results are shown in Figure 7. The DTA curve features a sharp endothermic peak at 73 °C, indicating that TNT undergoes a solid–liquid phase transition during melting. Meanwhile, the TGA curve indicates a 73.1% mass loss within a temperature range of 125 °C to 190 °C. This mass loss occurs at a significantly lower temperature than the known decomposition temperature of TNT, suggesting that the primary process during this stage is material evaporation rather than thermal decomposition. This conclusion is further supported by the flat endothermic peak observed in the DTA curve in the same temperature range, as the characteristics of the endothermic heat flow align closely with the thermal behavior typical of an evaporation process. Overall, the results from the TGA and DTA tests of TNT confirm that our MEMS cantilever multifunctional thermal analysis chip demonstrates significant advantages and wide applicability in the assessment of hazardous materials and energetic compounds.

## 7. Conclusions

In summary, this study successfully developed a joint thermal analysis technology that combines TGA and DTA using a MEMS-integrated cantilever. By incorporating thermocouples and resonant drive/detection resistors onto a polysilicon cantilever chip, we achieved the monolithic integration of both mass sensing and heat detection. The chip demonstrates technical advantages with a mass resolution of 5.5 picograms and a temperature resolution of 0.0082 °C, based on the mass sensing and differential thermal analysis functions confirmed by COMSOL (ver 5.0) simulations. This technology requires only nanogram-level samples to complete the measurements, overcoming traditional thermal analysis technology’s reliance on milligram-level samples. Experimental verification showed that the TGA-DTA curve obtained from the MEMS chip during the decomposition of nylon 6 in air aligns closely with data from traditional instruments, confirming the high-precision analytical capabilities of this miniaturized system. The chip is particularly well suited for the thermal analysis of hazardous materials, such as AP and TNT, due to the specific requirements for trace samples. By minimizing sample loading, it significantly reduces the risk of equipment explosion and the potential for contamination. Thermal analysis technology which incorporates miniaturization, high sensitivity, and low risk enhances both test efficiency and safety. Moreover, it offers innovative tools that overcome the limitations of traditional technologies in fields such as material science, catalysis, drug development, and hazardous substance analysis. As a result, it facilitates the expansion of thermal analysis methods into micro–nano-scales and extreme conditions.

## Figures and Tables

**Table 1:** Parameters of our work and other reported MEMS chips.

Function	Seebeck Coefficient	Power Response	Mass Sensitivity	Temperature Coefficient	Reference
DTA	2.9 mV/K	23 V/W	/	/	[17]
TGA	/	/	0.4 Hz/pg	1.9 ppm/K	[19]
DSC	4 mV/K	24 V/W	/	/	[21]
Mass sensing	/	/	0.01 Hz/pg	28.6 ppm/K	[29]
TGA	/	/	0.164 Hz/pg	20 ppm/K	[30]
Joint TGA/DTA	0.73 mV/K	6 V/W	0.09 Hz/pg	10.7 ppm/K	This work

## Data Availability

Data are contained within the article.
